# Paediatric atopic eczema (atopic dermatitis) in South Africa: A practical algorithm for the management of mild-to-moderate disease in daily clinical practice

**DOI:** 10.4102/safp.v62i1.5190

**Published:** 2020-11-23

**Authors:** Susanna M. Kannenberg, Sarah Karabus, Willem I. Visser, Jamilabibi Aboobaker, Magdalena M. Kriel, Michael Levin, Basil Magigaba, Ahmed Manjra, Rupesh Misra, Pholile Mpofu, Azwitamisi Tshigabe, Thomas Luger

**Affiliations:** 1 Division of Dermatology, Department of Medicine, Faculty of Health Sciences, Stellenbosch University, Cape Town, South Africa; 2 Division of Paediatric Allergology, University of Cape Town, Cape Town, South Africa; 3 Private Practice, Durban, South Africa; 4 Private Practice, Johannesburg, South Africa; 5 Private Practice, Port Elizabeth, South Africa; 6 Private Practice, East London, South Africa; 7 Department of Dermatology, Faculty of Dermatology, University of Münster, Münster, Germany

**Keywords:** atopic dermatitisd, atopic eczema, treatment algorithm, pimecrolimus, tacrolimus

## Abstract

**Background:**

Atopic eczema (AE) is a chronic, highly pruritic, inflammatory skin condition with increasing prevalence worldwide. Atopic eczema mostly affects children, impairing quality of life with poor disease control leading to progression of other atopic disorders. As most patients in South Africa have no access to specialist healthcare, a practical approach is needed for the management of mild-to-moderate AE in paediatric patients for daily clinical practice.

**Methods:**

A panel of experts in AE convened to develop a practical algorithm for the management of AE for children and adolescents in South Africa.

**Results:**

Regular moisturising with an oil-based emollient remains the mainstay of AE treatment. Severe AE flares should be managed with topical corticosteroids (TCSs). For mild-to-moderate AE flares in sensitive skin areas, a topical calcineurin inhibitor (TCI) should be applied twice daily from the first signs of AE until complete resolution. Topical corticosteroids may be used when TCIs are unavailable. In non-sensitive skin areas, TCSs should be used for mild-to-moderate AE, but TCIs twice daily may be considered. Proactive maintenance treatment with low-dose TCI or TCS 2–3 times weekly and the liberal use of emollients is recommended for patients with recurrent flares.

**Conclusions:**

This algorithm aims to simplify treatment of paediatric AE, optimising clinical outcomes and reducing disease burden. This approach excludes treatment of patients with severe AE, who should be referred to specialist care. Emphasis has been given to the importance of general skincare, patient education and the topical anti-inflammatory medications available in South Africa (TCSs and TCIs).

## Introduction

Atopic eczema (AE) is a chronic, relapsing, pruritic, inflammatory skin disease that is common during childhood.^1,2,3^ For both patients and their families, AE imposes a considerable burden and substantially impairs quality of life, particularly because of sleep disturbance as a result of itch.^1,2,4^ There is little available data regarding the incidence of AE in South Africa, although two phases of a global survey indicated that the majority of adolescents with AE from Cape Town have mild-to-moderate rather than severe disease.^5,6^ Evidence from a variety of sources suggests that the prevalence of AE is increasing in South Africa.^7,8^ From 1995 to 2002, increases were reported for adolescents (13–14 years old) in Cape Town for self-identified symptoms of AE (15.5% – 26.2%) and physician-diagnosed AE (9.6% – 16.7%).^7,8^ The prevalence of AE is increasing in tandem with the associated morbidity and economic burden of disease.^2,8^ Atopic eczema is more common in urban than rural settings, being found in 23.5% of 1–3 year old children in Cape Town and 1.8% of children in the rural Eastern Cape.^9^

The pathogenesis of AE involves epidermal barrier defects, immune abnormalities and environmental factors.^1^ Atopic eczema may be associated with concomitant sensitisation to food allergens in childhood and may be followed by progression to other atopic co-morbidities, such as allergic rhinitis and asthma.^10,11^

Children with moderate-to-severe, therapy-resistant AE are at high risk of food allergy. Approximately 40% of patients attending a tertiary-level dermatology referral clinic in Cape Town had concomitant immediate type immunoglobulin E-mediated food allergies, with egg and peanut being the most common.^12^

Atopic eczema is increasingly being regarded as a systemic disease, which may be associated with a range of non-atopic co-morbidities such as cardiovascular disease and metabolic diseases, as well as psychological disorders (e.g. attention-deficit hyperactivity disorder), the pathophysiology of which may involve underlying long-term systemic inflammation and/or chronic sleep deprivation.^13,14,15,16^ Optimal management of mild-to-moderate AE in paediatric patients requires a multifaceted approach that combines the education of patients and caregivers with a treatment approach that involves emollients, topical corticosteroids (TCSs) and topical calcineurin inhibitors (TCIs).^3,17,18^ Atopic and non-atopic co-morbidities should also be treated.

This article proposes a practical approach for the management of mild-to-moderate AE and acute flares in paediatric patients (children and adolescents) in South Africa. The intention is that the information presented will supplement existing international and national, evidence-based treatment guidelines for use by general practitioners in daily clinical practice, as well as dermatologists, paediatricians and nurse practitioners. The focus of this information is the management of mild-to-moderate AE and acute flares, rather than severe disease.

## Methods

A panel of 12 experts in AE was formed to develop a practical algorithm for the management of this disease in daily clinical practice for children and adolescents in South Africa. Professor Luger proposed the first draft of the algorithm, which was discussed with the other authors and adapted to South Africa based on their expertise, local knowledge, guidelines^19,20,21,22,23,24,25^ and relevant literature published up to 2018. The initial algorithm proposed by Professor Luger has also been adapted for the Middle East by a separate group of experts.^26^

### Practical management algorithm for paediatric atopic eczema in South Africa

**FIGURE 1:**
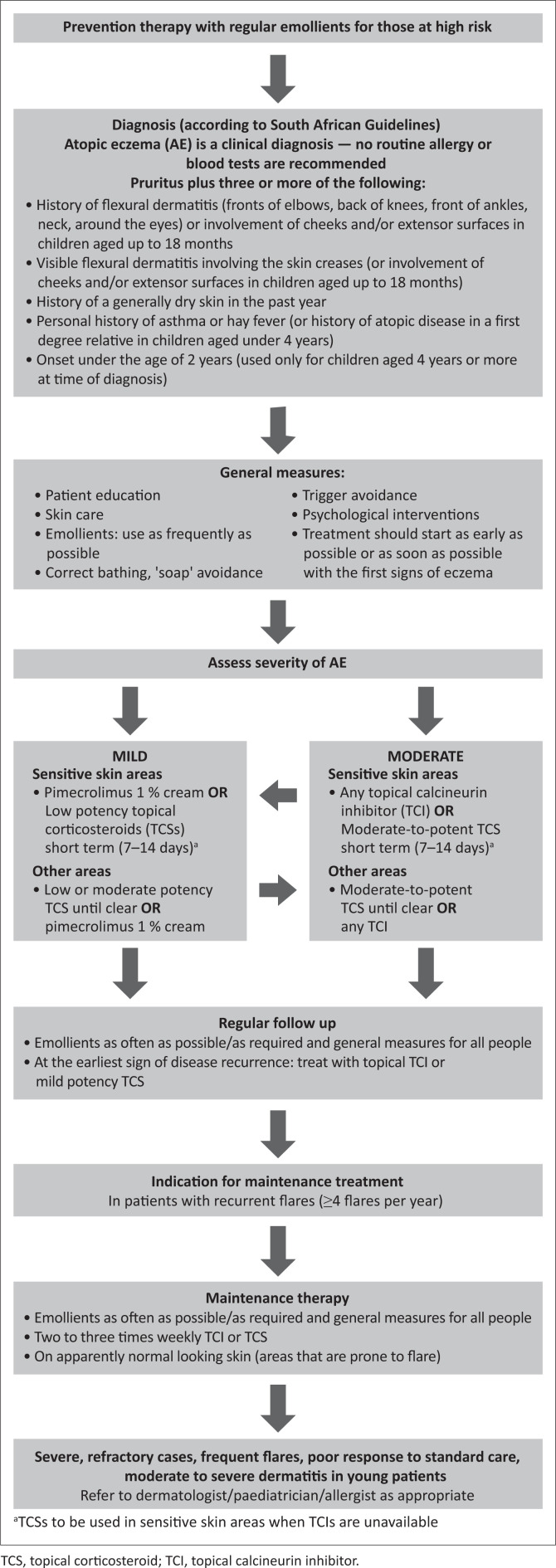
Treatment algorithm for paediatric patients with atopic eczema (AE; atopic dermatitis) in South Africa.

A treatment algorithm for paediatric patients with AE in South Africa is shown in Figure 1.

### Prevention measures

Epidermal barrier dysfunction, impaired water retention and allergic sensitisation are thought to be important in the onset of AE.^27,28,29^ Moisturising the skin twice daily from birth with a bland emollient may therefore be beneficial in those at high risk of developing AE. ‘High-risk patients’ are defined as ‘having a parent or full sibling who has [or had] physician-diagnosed atopic dermatitis, asthma or allergic rhinitis’.^27^ However, this approach has not been supported by other studies.^30^ There is no high-quality evidence for the prevention of the development of AE regarding the effectiveness of pre- or probiotics,^22^ hypo-allergenic infant formula^31^ or the avoidance of certain foods by pregnant and breastfeeding women.^22,32^

## Diagnosis

Atopic eczema is primarily a clinical diagnosis. The current South African guidelines^33^ dictate that criteria for diagnosis of AE must include pruritus and three or more of: a history of flexural dermatitis; visible flexural dermatitis; history of dry skin in the past year; a personal history of asthma or hay fever (or history of atopic dermatitis in a first-degree relative aged <4 years); or onset under the age of 2 years.^33^ A detailed clinical history should include: age of onset; personal and family history of atopy; pattern and severity of symptoms; frequency of itch with a focus on affected body areas; response to previous or current treatments; possible irritants or allergic triggers; dietary history; growth and development; and the psychosocial impact that AE has on the child and their parents and caregivers.^34^

### Diagnosis of atopic eczema in infants

The anatomical and pathophysiological characteristics of AE in infants are typically different from those observed in older children.^17^ In infants, AE begins after the first weeks of life and presents in facial, scalp and extensor regions.^17^ This contrasts with older children, in whom flexural involvement predominates.^17^ South African guidelines (for all ages)^33^ recommend diagnosis of AE according to the original UK Working Party criteria.^35^ However, these criteria cannot be applied to infants^19^ and consequently, revised versions of the UK Working Party criteria^36,37,38^ or the Hanifin and Rajka criteria^39^ should be applied.

### Diagnostic considerations in South Africa

Poor access to healthcare and prioritisation of other infectious diseases have led to under-reporting of allergic diseases such as AE in Africa.^40^ In rural settings, healthcare providers may be in short supply and have poor knowledge of allergic conditions.^41^ As such, in these areas, ‘teledermatology’ may improve access to healthcare.^41^ Another consideration is that allergen tests that have been developed in Europe may be unsuitable for African patients, who may have different allergen sensitivities than European patients.^40^

## Assessment of severity

A holistic approach is important when assessing the severity of AE and the impact it has on a child’s quality of life, which are directly linked.^34^ The severity of AE may be assessed using the Investigator’s Global Assessment or, more accurately, with either the SCORing Atopic Dermatitis index or Eczema Area and Severity Index score.^42,43^ Mild AE can be defined as areas of dry skin with infrequent itching (with or without small areas of redness), which have a minor impact on everyday activities, sleep and psychosocial well-being. Moderate AE involves areas of dry skin, frequent itching and redness with possible excoriation and localised skin thickening, which have a moderate impact on everyday activities and psychosocial well-being and may result in disturbance of sleep. Severe AE is associated with widespread areas of dry skin, incessant itching and redness, and can involve excoriation, extensive skin thickening, bleeding, oozing, cracking and altered skin pigmentation. This level of physical severity causes severe limitation of everyday activities and psychosocial functioning, as well as regular loss of sleep.^44^

Active secondary bacterial infection must be excluded during the assessment of AE as an acute flare often presents with crusts and oozing, which may closely resemble an acute bacterial infection.^34^ In addition, ethnicity should be accounted for, as black African, Afro-Caribbean and Asian children may present with atypical features, namely extensor surface involvement rather than flexural and perifollicular accentuation.^45^

## Treatment goals

The overall goals of treatment for AE are to reduce the signs and symptoms of disease, induce disease remission and improve the quality of life of both patients and their caregivers.^23^ Treatment options range from general daily management with emollients to topical anti-inflammatory medication, depending on the severity and frequency of symptoms. Most cases of AE can be treated clinically; however, patients should be referred to a dermatologist, paediatrician or allergist if they have: moderate-to-severe AE; severe, refractory AE; frequent flares; a history of concomitant allergic manifestations including immediate-type food allergy; or a poor response to treatment.^46^

## General recommendations for atopic eczema management

### Emollient use

In agreement with evidence-based guidelines for AE management in South Africa,^33^ we recommend that all patients should use an emollient regularly as maintenance therapy. Emollients should be applied over the entire body, in single strokes, in the direction of hair growth at least twice a day.^47^ The choice of emollient is dependent on the patient’s preference, but should be free from fragrances and colorants.^23,48^ If cost is a limiting factor, petroleum jelly or glycerine mixed with petroleum may provide effective and affordable alternatives to standard emollients.^49^

### Bathing and hygiene

Bathing once a day in lukewarm water helps to hydrate and cleanse the skin by removing crusts and/or scales, but bathing time should be limited to 5 min to prevent the skin from drying out.^24^ Bath additives should be non-irritating and shampoo should be specifically indicated for AE.^24,34^ The routine use of household antiseptics or soaps is discouraged and a non-soap cleanser, such as aqueous cream or an emulsifying ointment, should be used.^24,50^ After bathing it is best to gently pat, rather than rub, the skin dry and then moisturise the entire body.^24^ Aqueous cream should not be used as a leave on product as this has been shown to have a negative impact on the skin barrier.^51^

### Additional measures

Additional measures include: keeping the patient’s nails short, to prevent scratching and secondary infection^50^; not overdressing to keep the skin cool; and using cotton clothing, which is less irritating than clothes made from wool and other rough fibres.^52^ Non-irritating sun protection products should be used and smoking should be avoided near children, as environmental tobacco smoke is a known trigger for acute exacerbations of AE.^53^ Routine testing for food allergies or food avoidance is not recommended unless there is a clear indication^24,54^; specific food avoidance can lead to nutritional deficiencies, exacerbate anxiety in both child and family and may also contribute to the development of a food allergy.^54,55^ Food allergies should be treated by a specialist and are beyond the scope of this article.

### Patient and caregiver education and support

In South Africa, a high proportion of patients use complementary and alternative therapies, which lack high-quality evidence in support of their efficacy.^56^ Patient education is therefore of particular importance. Nurse practitioners should be educated in childhood AE and trained to provide evidence-based health education to patients and caregivers.^57,58,59^

Before therapy begins, it is best to offer either educational material or courses on AE treatment to patients and their caregivers, which should include information regarding common triggers of AE, aspects of basic skin care and potential side effects of treatment.^33,50,54^ Patients and caregivers who are informed by educational courses have higher quality of life as they can anticipate side effects, which reduces the likelihood of premature treatment discontinuation.^18^ Patients should also be provided with psychological support to improve emotional well-being, although there is a lack of high-quality evidence supporting the efficacy of psychological interventions.^33,60,61^

The Allergy Foundation of South Africa (www.allergyfoundation.co.za) has patient-centred, downloadable information materials that may be of use, including a general pamphlet on AE,^62^ an eczema action plan^63^ and a leaflet on wet-wraps.^64^ Wet-wrap clothing (rather than bandages) is now available in South Africa, simplifying this important treatment method.^65^ Providing patients and caregivers with this information should be prioritised and is as important as other treatment strategies.^66^

Communication with patients is important as a regular follow-up improves the clinical outcome through greater adherence to treatment.^67^ Any change in the treatment plan should be clearly explained to the patient.

## Topical anti-inflammatory medication: Corticosteroids and calcineurin inhibitors

Active, topical anti-inflammatory treatment should be started at the first sign of a flare and be applied on hydrated skin, particularly when using ointments.^24^ Topical corticosteroids and TCIs are the anti-inflammatory medications of choice to treat AE (Table 1).^68^

**TABLE 1:** Topical calcineurin inhibitors and topical corticosteroids.^68^

Treatment	Formulation	Activity¶	Efficacy	Key side effects	Position in algorithm
Pimecrolimus 1% †,‡	Cream	Acute: + Chronic: +++	Short- and long-term treatment	Application site reactions (e.g. burning, erythema and pruritus)	*Acute mild-to-moderate AE* TCIs as first-line therapy for sensitive areas and a therapeutic option in other body locations *Maintenance therapy*
Tacrolimus 0.03% and 0.1% †,§	Ointment	Acute: + Chronic: +++	Short- and long-term treatment	Application site reactions (e.g. burning, erythema and pruritus)	*Acute moderate AE* TCIs as first-line therapy for sensitive areas and a therapeutic option in other body locations *Maintenance therapy*
TCSs	Creams and ointments	Acute: +++ Chronic: +++	Short-term treatment	Skin atrophy Epidermal barrier impairment	*Acute severe AE flares* First-line, short-term treatment until symptom improvement *Acute mild-to-moderate AE* TCSs are an option for mild-to-moderate AE, except for sensitive body sites (if TCIs are available) *Maintenance therapy* When TCIs are not an option

AE, atopic eczema; TCI, topical calcineurin inhibitor; TCS, topical corticosteroid.

† , Limited availability in government sector tertiary and quaternary hospitals in South Africa.

‡ , Registered in South Africa for use in mild-to-moderate AE in ages 2 years and older. Expert opinion recommends safety in children less than 2 years of age.

§ , 0.03% indicated for use in children aged 2 years and older; 0.1% indicated for adolescents aged 16 years and older. Tacrolimus is indicated for moderate-to-severe AE in South Africa.

¶ , Activity key: ‘+’, effective, ‘++’, very effective, ‘+++’, highly effective.

### Topical corticosteroids

A list of the TCSs that are available in South Africa are shown in Table 2.^69^ The costs to use these steroids range from 2.21 (1% hydrocortisone acetate cream) to 17.63 (0.1% methylprednisolone aceponate scalp solution) South African rand (ZAR) per gram.^70^ Topical corticosteroids have been shown to quickly and effectively control flares associated with acute AE.^3^ There are few side effects for patients when TCSs are chosen appropriately and used in the short term, but local effects may occur when TCSs are used for extended periods of time or at an inappropriate strength.^24,71^ These side effects may include striae, skin atrophy, purpura, telangiectasia and impaired epidermal barrier function.^71^ Rare systemic side effects of TCSs include hypothalamic–pituitary–adrenal axis suppression and Cushing’s syndrome.^71,72^ The systemic side effects of TCSs are of particular concern in children who have a higher surface area-to-body weight ratio, which may lead to increased systemic absorption.^71^ Sensitive skin areas, such as the face, eyelids and skin flexures, may be at particularly high risk of developing side effects when TCSs are misused.^71^

**TABLE 2:** Topical corticosteroids available in South Africa.^69^

Treatment	Formulation
**Lowest potency**	
0.5% hydrocortisone acetate	Cream, ointment
**Low potency**	
1% hydrocortisone acetate	Cream, ointment
**Moderate potency**	
Beclomethasone diproprionate Clobetasone butyrate Fluticasone propionate Hydrocortisone 17-butyrate Methylprednisolone aceponate Momethasone furoate	Cream Cream Cream, ointment Cream, lipocream, ointment, lotion, emulsifying lotion Milk, scalp solution, cream, ointment, fatty ointment Cream, ointment, lotion
**Potent**	
Betamethasone valerate Fluocinolone acetonide Diflucortolone valerate	Cream, ointment, solution, scalp solution Cream, ointment, gel Cream, fatty ointment, forte ointment
**Very potent**	
Clobetasol propionate Betamethasone dipropionate	Cream, ointment, shampoo, scalp solution Cream, ointment

A growing fear of side effects related to TCSs amongst both patients and healthcare providers, or ‘corticophobia’, has led to widespread lack of adherence and underprescribing of TCSs in recent years. A questionnaire administered to patients in the United Kingdom in 1998 determined that 72.5% experienced corticophobia whilst 24% were non-adherent to corticosteroid treatment.^73^ The results of another survey of patients in France, published in 2011, found 80.7% of patients experienced corticophobia whilst 36% were non-adherent.^74^ This lack of adherence is a major contributor to TCS treatment failure and persistently uncontrolled AE.^73,74^ Educating patients on the correct use of corticosteroids (i.e. the various potencies and correct dosing) and the low risk of side effects associated with appropriate use is therefore critical to improve adherence and to avoid undertreatment.^20,24^ If corticophobia is significantly impacting treatment outcome, a referral should be made to a specialist for dermatological advice.^33^

### Topical calcineurin inhibitors (pimecrolimus and tacrolimus)

In South Africa, pimecrolimus and tacrolimus are indicated for the treatment of mild-to-moderate and moderate-to-severe AE, respectively.^33^ Although TCSs are recommended as first-line treatment for most patients because of their superior efficacy, in the American Academy of Dermatology guidelines TCIs are recommended as first-line treatment for patients with AE in sensitive body areas, patients with steroid-induced atrophy and patients who have current long-term uninterrupted topical steroid use.^20^ In the European AE guidelines, pimecrolimus is recommended for facial lesions and for children, and both pimecrolimus and tacrolimus for long-term maintenance treatment.^24^

Patients should be informed before treatment begins that application-site erythema and/or a burning sensation are frequent side effects of TCI treatment, but these usually diminish after 3–4 days of use.^68^ These effects may be managed by the systemic use of a non-steroidal anti-inflammatory, such as acetylsalicylic acid, 1 h before application of the product for the first 3–4 days.^75^ Placing the TCI medication in the fridge may also be of help.^76^ In studies with paediatric patients, local burning sensation was experienced by up to 36% of patients treated with tacrolimus and 7.4% of patients treated with pimecrolimus.^77^ Since 2006, TCIs have carried a controversial boxed warning based on a theoretical risk of skin and lymphoma malignancy,^78^ leading to hesitancy for long-term prescription, but this perceived risk has not been supported by rigorous post-marketing surveillance. In a recent systematic review of the last decade of clinical experience, the authors found that there was no evidence that use of TCIs is associated with an increased risk of malignancy.^78^

In a network meta-analysis of clinical studies involving 19 treatment comparisons and 6413 children with AE, pimecrolimus 1% cream and tacrolimus 0.03% and 0.1% ointments were found to have similar efficacy and safety for the treatment of paediatric AE.^79^ Similarly, in a comparative study of both TCIs in 141 children and adolescents with moderate AE, pimecrolimus 1% cream and tacrolimus 0.03% ointment were shown to have similar efficacy for the treatment of AE in different body locations (other than sensitive areas) over 6 weeks.^68^

Topical calcineurin inhibitors are not approved for use in infants (age ≤ 2 years) with mild-to-moderate AE in South Africa.^33^ However, extensive evidence from nine studies that were conducted in more than 6700 patients (including 4799 infants and 1312 children) supports the clinical efficacy and safety of pimecrolimus 1% in infants.^80,81,82,83,84,85,86,87,88^ The results of an open-label phase 2 study in 50 infants suggest that tacrolimus 0.03% is also efficacious in this age group, with similar tolerability as in older children.^89^

When prescribing topical medication, it is important to balance efficacy and side effect profiles. Pimecrolimus has been shown to be as effective as a mild-to-medium potency TCSs^86^ but without inducing TCS-related side effects, such as visible skin atrophy and barrier dysfunction.^90,91,92^ Furthermore, paediatric studies have demonstrated that pimecrolimus helps in cases where corticophobia is affecting adherence to therapy.^82,86,87,93,94^

## Recommendations for pharmacological management of atopic eczematous flares

### Mild-to-moderate atopic eczema flares in non-sensitive skin areas

For mild–acute AE flares in paediatric patients, we recommend low or moderate potency TCSs (Table 2) once daily or pimecrolimus twice daily until clear. For moderate AE flares, patients may use an appropriate strength TCSs, either once or twice daily or a TCI twice daily. The potency of TCS should be selected based on the severity of AE. The medication should be applied to all affected areas at the first signs and symptoms of an acute flare up until complete resolution of these symptoms. These signs and symptoms may include transient erythema, bumps, warmth, skin thickening, itching or tingling. If a TCI is selected as first-line therapy and the acute flare is not controlled, a short (7–14 days) course of TCS may achieve control, which can then be gradually reduced or switched to a TCI to maintain results.

### Mild-to-moderate atopic eczema flares in sensitive skin areas

We recommend a TCI twice daily for mild-to-moderate AE flares in children and sensitive body areas. In the European AE guidelines, pimecrolimus is recommended as the treatment of choice for facial lesions and children.^24^ The treatment should be applied twice daily from the first appearance of signs and symptoms until complete resolution.

### Severe atopic eczema flares

For the management of severe disease flares, moderate or potent TCSs are recommended (Table 2) as they have been shown to effectively and rapidly control disease flares in paediatric patients with AE.^20,95^

**FIGURE 2:**
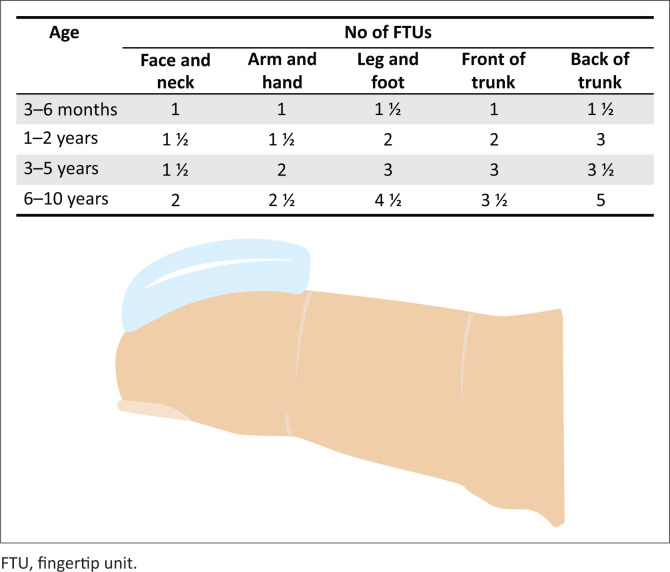
‘The fingertip method’. The amount of fingertip units (the amount of ointment or cream covering the area on the finger from skin-crease to the tip of an adult’s index finger) required for steroid application depends on the age of the child and the specific body area that needs to be treated.^50^

Prior to initiating TCS treatment, clear instruction must be given regarding the safe use of the TCS. This guidance may include: the potency of TCS to be used and where on the body it should be applied; frequency of use; the correct amount of cream or ointment to be used per body area as per the ‘fingertip method’ (Figure 2)^50^; avoiding application in sensitive areas such as eyelids, which are more prone to the development of side effects; and washing hands after use (unless the hands themselves are affected). Side effects should be discussed, but it is important to emphasise that these effects are unlikely with short-term usage in the prescribed manner.^73,74^ Should tachyphylaxis occur during TCS treatment, an alternative, more potent preparation can be tried if necessary.^71^ Cases of severe, chronic AE should be referred to a specialist.^46^

## Maintenance treatment

For patients with four or more acute flares per year, we recommend proactive maintenance treatment. This is a combination of long-term, low-dose anti-inflammatory treatment (TCIs or TCSs of the appropriate strength) 2–3 times weekly, applied to areas of skin previously affected by AE, together with the liberal use of emollients over the entire body. Alternatively, intermittent therapy with TCI may be recommended which involves the resumption of treatment at the very first signs of a new flare, that is, pruritus. Currently, there is no evidence to suggest that the long-term management of AD with proactive therapy is superior to management with intermittent therapy. Studies have shown that using TCIs or TCSs as maintenance therapy in addition to emollients is significantly more effective at controlling relapses compared with emollient use alone.^96,97,98,99,100^ The duration of maintenance therapy is determined on an individual basis. Discontinuation may be attempted by slowly decreasing the frequency of application and closely monitoring the results.

## Conclusion

The prevalence of paediatric AE in South Africa is increasing in tandem with the associated clinical, psychosocial and economic burden of the disease. There is a need for effective, evidence-based treatment strategies for managing AE, which should be tailored to patient preference to ensure adherence. General skincare with gentle bathing regimes and regular application of the appropriate emollients is a vital consideration in the effective management of AE. When mild-to-moderate AE flares occur and no patient preference has been expressed, consideration should be given to the choice of TCSs or TCIs as a topical anti-inflammatory. Topical corticosteroids should be used for mild-to-moderate AE on non-sensitive skin areas, but TCIs may be preferable for treatment of sensitive skin, when side effects of TCSs have occurred or where TCSs are already being used on a continuous basis. For patients with more severe flares, TCSs may achieve control followed by weaning to the lowest possible dose, emollients alone or TCI maintenance therapy. For patients with regular (≥ 4 per annum) acute flares, we recommend proactive maintenance treatment with a TCI or TCS 2–3 times weekly. The approach presented here is intended to simplify the treatment of paediatric mild-to-moderate AE in daily practice in South Africa, thereby optimising clinical outcomes and reducing the burden of disease.
